# When superfluidity meets superconductivity in the extraction of ^3^He isotope from liquid helium

**DOI:** 10.1038/s41598-025-05542-8

**Published:** 2025-07-02

**Authors:** Wojciech Kempiński, Piotr Banat, Mateusz Kempiński, Zbigniew Trybuła, Jakub Niechciał, Maciej Chorowski, Jarosław Poliński, Katarzyna Chołast

**Affiliations:** 1 Institute of Molecular Physics, Polish Academy of Sciences, 60-179 Poznań, Poland; 2 Faculty of Physics and Astronomy, Adam Mickiewicz University in Poznań, 61-614 Poznań, Poland; 3 Department of Cryogenics and Aerospace Engineering, Wroclaw University of Science and Technology, 50-370 Wrocław, Poland; 4 ORLEN SA Polish Oil and Gas Branch in Odolanow, 63-430 Odolanów, Poland

**Keywords:** Superfluidity, Superconductivity, Helium-3, Isotope separation, Energy science and technology, Materials science, Physics

## Abstract

The world needs clean energy. One of the most promising ways of producing it in large amounts is the helium3-deuterium (^3^$\hbox {He-D}$) fusion reaction. Although there are numerous sources of ^3^$\hbox {He}$ on Earth, most of them are either difficult to access or unprofitable to operate. The main problem underlying the shortage of ^3^$\hbox {He}$ is the lack of an effective method of obtaining this isotope. Here we report the results of quantum filtration of ^3^$\hbox {He}$ from liquid helium in a superfluid state (below the $\lambda$-transition), with the use of an entropy filter made of a high-temperature superconductor YBCO-123. During the operation of so-called *fountain effect* generated with this filter, unlike the other filters, we observed a strong increase of ^3^$\hbox {He}$ concentration downstream, where only pure $^{4}\hbox {He}$ was expected. This effect occurred due to the unique combination of two quantum phenomena—superfluidity and superconductivity, leading to the observation of a low-temperature rectification-like process. Rectification of helium isotopes does not require lowering the temperature below the $\lambda$-transition, so the process can be more economical than filtration. Moreover, micro-superconductors could be applied also to the extraction of deuterium, thus allowing the same method to be used for both crucial components of the ^3^$\hbox {He-D}$ fusion. This method should be easy to upscale and could be used in space (with less energy input) as ^3^$\hbox {He}$, the crucial isotope for future energy, is also sought beyond the Earth.

## Introduction

As a matter of great importance for the clean energy problem, the deep shortage of ^3^$\hbox {He}$ isotope^1^ was reported to the American Congress^2^ where, among other possibilities, the extraction of the ^3^$\hbox {He}$ from the liquid helium was considered. Amongst many types of fusion reactions, those involving ^3^$\hbox {He}$ isotope are of particular interest^3–8^. The third-generation fuel ^3^$\hbox {He}$+^3^$\hbox {He}$ may seem very attractive due to zero waste, however, the much lower plasma ignition temperature of reaction D+^3^$\hbox {He}$ $\rightarrow$ p(14.7 MeV) + $^{4}\hbox {He}$(3.7 MeV) makes it more viable, despite some radioactive waste coming from parasitic reactions (e.g. D+D). The unique possibility of harvesting the high energy of protons via the field reversed configuration (FRC) technique^6,9^ makes this reaction even more interesting. Currently, ^3^$\hbox {He}$ is mostly acquired from radioactive material storage sites (e.g. nuclear warheads). But, as the demand for ^3^$\hbox {He}$ increases, new stable sources are needed. The search for ^3^$\hbox {He}$ has been shifted to Earth’s nearest surroundings, the Moon, as well as to other planets of the solar system, that have no protective magnetic field^10^. The exploitation of these supposedly rich, but very distant sources, will require an enormous financial outlay and as such is going to be seriously stretched not only in space but also in time^8^. One of the most promising methods to obtain significant amounts of ^3^$\hbox {He}$ on Earth is extraction from natural gas, especially if the process is integrated into the liquid helium production cycles of the existing low-temperature natural gas purification plants^2^. There are many such installations in the world and the building of new ones is also being planned^11,12^. It needs to be pointed out that the separation must be performed before helium is put to any use, otherwise the ^3^$\hbox {He}$ isotope would be irretrievably lost.

## Methods

### Helium and entropy filter

**Fig. 1:**
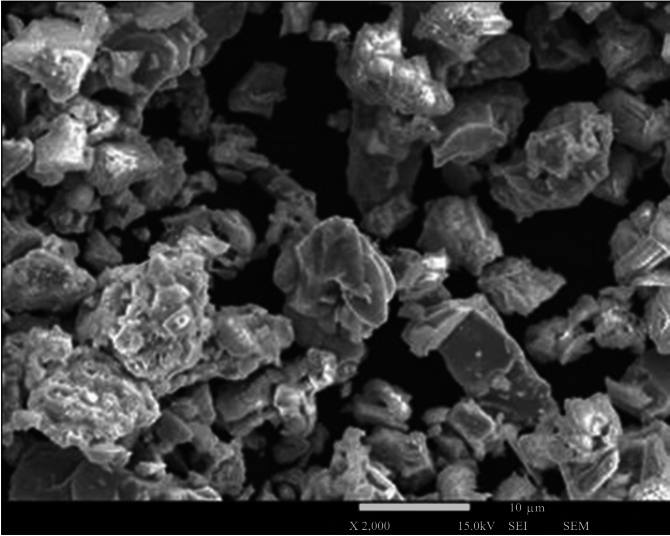
SEM image of YBCO-123 micro-sized powder. The image was obtained with Jeol 7001TTLS scanning electron microscope, at 15 kV accelerating voltage with the use of a secondary electron detector. For better visibility, YBCO-123 granules were dispersed on a silicon substrate.

The liquid helium used in the experiment came from Polish sources and was supplied by the Polish Oil and Gas Company—Odolanów plant. The concentration of ^3^$\hbox {He}$ in helium from these sources was in the range of 0.15–0.25 ppm. The concentration of $^{4}\hbox {He}$–^3^$\hbox {He}$ mixtures was measured with the QMS 700 mass spectrometer produced by Pfeiffer Vacuum GmbH, which allowed for the detection of mass at the $10^{-3}$ ppm level. QMS 700 parameters are: mass range 1–128 amu, sensitivity—single ion $10^{-19}$ A, resolution—one neutron. Entropy filters are usually made of fine-grained materials such as corundum^13,14^ although other powders can be used as well^15^. In this work, we used a superconducting fine-grained YBCO-123^16^ with a mean grain size of approx. 10 μm, produced by Can Superconductors s.r.o. Czech Republic—https://www.can-superconductors.com/. Figure 1 shows a scanning electron microscopy image of YBCO-123.

### Filter efficiency

**Fig. 2:**
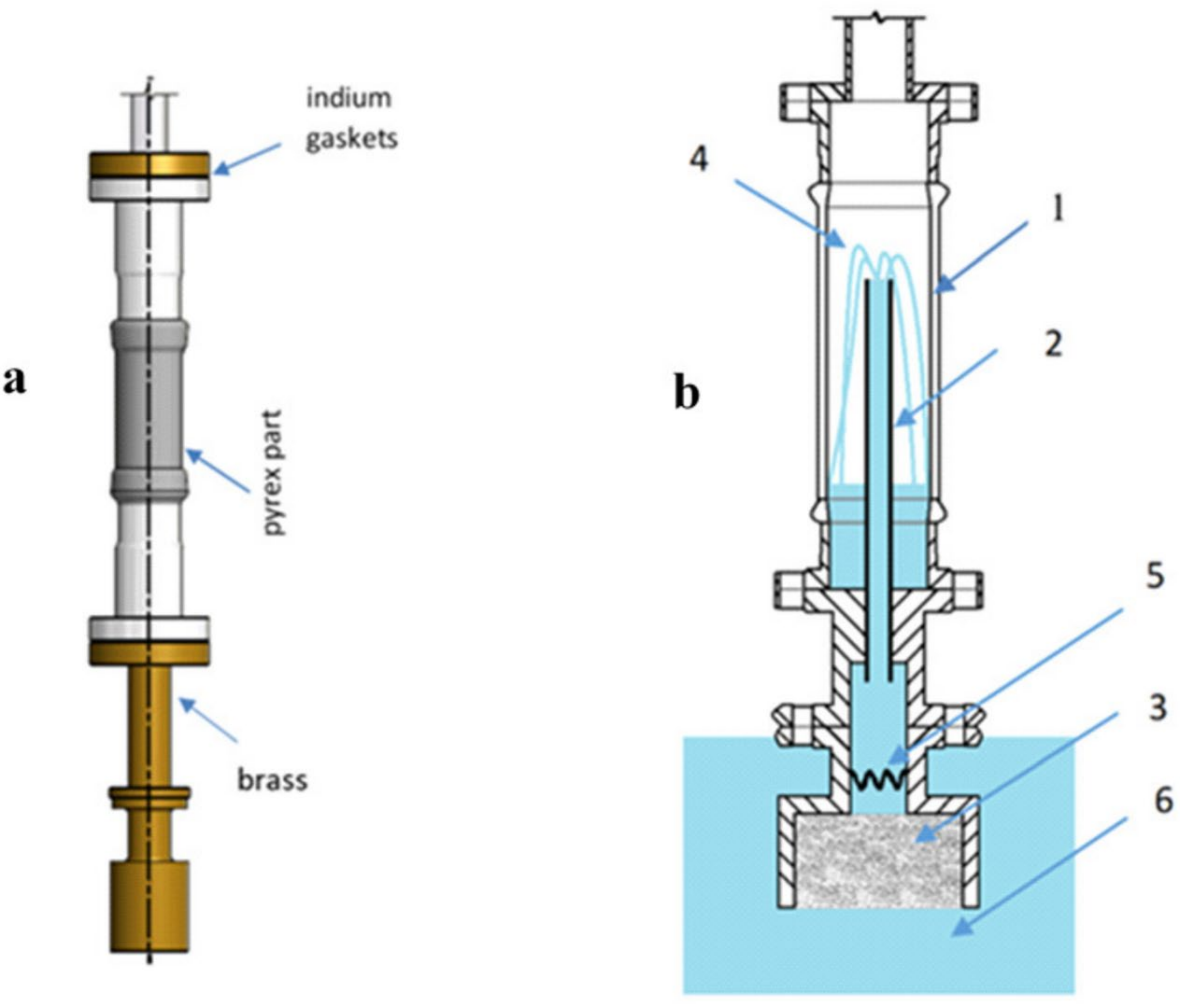
Construction of the glass cryostat insert for efficiency measurements. (**a**) External view of the device with an indication of the materials used. (**b**) Schematic representation of the device’s interior. Pyrex part 1 of known volume allows direct observation of superfluid component transfer through the capillary 2 and measures the efficiency of YBCO-123 filter 3 via the fountain effect 4 generated by the heater 5 immersed in superfluid helium 6. The heater is the resistor: 128 ohms at RT and 124 ohms at 4.2 K, placed in a downstream direction. 3D model created in Autodesk Inventor Professional 2012 (https://www.autodesk.com/products/inventor/overview).

Filter efficiency was registered via the volumetric flow, which required strict control of the *fountain effect* (see Supplementary Material—Movie 1). It was done with a special insert that was placed inside the helium cryostat, allowing for the direct observation of the fountain effect—see Fig. 2. The working structure is shown in the two movies (see Supplementary Material). For better visibility, these short films are presented using carbon nanotube filters^15^.

### Filter effectiveness

The experiment was performed using a 25-liter bath cryostat equipped with a heat exchanger. The setup and the cooling process were described in detail in^17^. The cooling power of the heat exchanger was stabilized step by step up to a maximum of 700 mW. Samples were taken up and downstream of the filter at the same time and concentrations were measured with the mass spectrometer. In the cooling process, about 7–8 liters of helium were lost, so about 18 liters of $^{4}\hbox {He}$+^3^$\hbox {He}$ mixture were involved in the filtration process in a single experiment.

## Results

### Quantum filtration with the use of the entropy filters

Effective ^3^$\hbox {He}$ separation from $^{4}\hbox {He}$–^3^$\hbox {He}$ mixture cannot be performed by standard membrane-based methods, as the atoms of both isotopes are of the same size. Thus, the filtration must utilize entropy filters that operate at low temperatures, below the lambda transition, $\hbox {T}_{\lambda }=2.17(68)$ K, where quantum effects come into play. At $\hbox {T} < \hbox {T}_{\lambda }$ $^{4}\hbox {He}$ forms a Bose-Einstein condensate and is considered superfluid. In this state separation of $^{4}\hbox {He}$ and ^3^$\hbox {He}$ occurs via the thermomechanical effect, also known as the *fountain effect* or thermomechanical pumping^13,18–20^. The *fountain effect* allows the superfluid component to be effectively transported through the entropy filter in the downstream direction, as a heater placed close to the filter generates osmotic-like pressure driving the thermomechanical pump. ^3^$\hbox {He}$ as a part of a normal component (that becomes superfluid far below ${\text{T}}_{\lambda }$ at the mK level^19^) is stopped by the entropy filter, and the mixture in front of this filter (upstream direction) becomes ^3^$\hbox {He}$-enriched. The first experiments using the above process together with the rectification method were described by Kuznetsov^21^ and the Eselson group^14^. Our general approach focuses on the use of functional micro- and nanomaterials to create entropy filters, whose specific physical properties might affect the filtration. In this work, we employ micro-sized YBCO-123 which becomes superconducting below temperature ${\text{T}}_{c}$ = 92 K and compare it to the classical entropy filters^13,15,17^. In our experiments, we used liquid helium with $\sim$ 0.2 ppm of ^3^$\hbox {He}$^ 2^, to show that quantum filtration can be employed even for very low starting ^3^$\hbox {He}$ concentrations, which is common in the liquid helium obtained from natural gas deposits.

Two essential parameters of the entropy filters were examined: efficiency and effectiveness.

### Efficiency of entropy filters

**Fig. 3:**
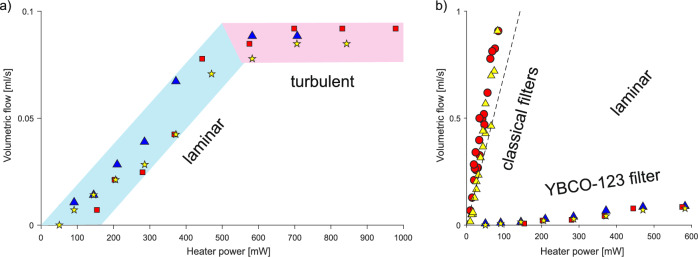
Volumetric flow of helium versus heater power describing the filter efficiencies: (**a**) two characteristic regions laminar and turbulent are shown for YBCO-123 filter and presented as the three measured series—blue triangles, red squares, and yellow stars. (**b**) comparison of laminar regions for classical and superconducting entropy filters. The slopes define individual efficiencies: red circles and yellow triangles are taken from^15^ together with the data from Nakai’s paper—the dashed line (the average of the values from^13^). Red squares, blue triangles, red squares, and yellow stars represent three series of YBCO-123 filter.

The slope of the dependence of volumetric flow on the power of the heater, used to activate the thermomechanical pumping, can be taken as a measure of the filter’s efficiency (see Supplementary Material—Movie 2). Data for YBCO-123 filter efficiency tests are presented in Fig. 3a. The obtained result suggests that the superconductor works similarly to any other fine-grained materials^13–15,17,21^. Volumetric flow increases linearly in the laminar superfluid region, saturating when the turbulent flow appears due to the critical heat input^13^. Significant differences occur when comparing the efficiency of YBCO-123 filter with the efficiencies of the classical ones. For different types of classical filters shown in Fig. 3b, the process started at a very low power level and the efficiencies were comparable. Quite different behavior was observed in the case of the superconducting YBCO-123 filter. The fountain appeared only for the relatively high initial power of 50 mW, and the slope was drastically lower in comparison to the classical filters. This unexpected behavior can be explained based on the significantly different thermal properties of two quantum systems that meet in the experiment: superfluid helium, whose thermal conductivity is extremely high^22^, and superconducting YBCO-123 with very low thermal conductivity that drastically drops below 50 K, down to about 1 $\hbox {Wm}^{-1}\hbox {K}^{-1}$ at 2 K^16,23,24^.

Normally, when the heater is on, the temperature of classical entropy filters quickly increases, as the superfluid medium transfers heat very effectively. The superconductor-based filter requires much more time (or energy) due to its low thermal conductivity. So, the thermomechanical pumping requires more heater power to start. This is why the initial heater power for the superconducting filter must be higher and its efficiency is much lower than in the classical case.

### Effectiveness of entropy filters

**Fig. 4:**
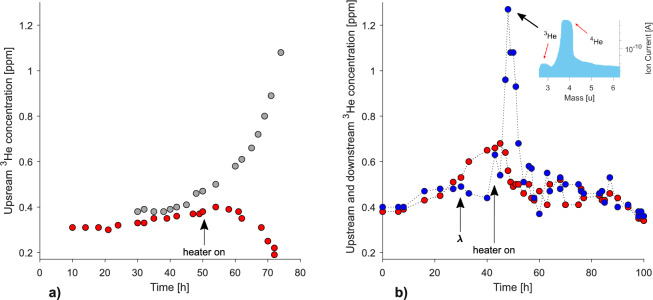
Upstream and downstream concentrations of ^3^$\hbox {He}$ as a function of the filtration time: (**a**) upstream concentration for YBCO-123 (red circles) as compared to MWCNTs (gray circles—data taken from^15^ and shifted in time for better visualization) at 55 mW heater power. (**b**) upstream (red circles) and downstream (blue circles) ^3^$\hbox {He}$ concentrations measured simultaneously, black arrows mark the appearance of the lambda transition and switching on the heater to drive the fountain effect. Insert shows the mass spectrum with additional arrows indicating the ^3^$\hbox {He}$ and $^{4}\hbox {He}$ concentrations.

The constant amount of helium was used for filtering in this part of our experiment (see “Methods” ). Thus, the final concentration of ^3^$\hbox {He}$ in the $^{4}\hbox {He}$+^3^$\hbox {He}$ mixture depended only on the effectiveness of the filter and was considered to be its measure. The filtration was performed in the laminar flow regime, at 70 mW heater power. The result (Fig. 4a—red circles) was compared with the effectiveness of the MWCNTs-based filter (Fig. 4a—gray circles) taken from^15^. Instead of a smooth increase, characteristic of the previously known classical filters, the concentration of ^3^$\hbox {He}$ upstream started to decrease (red circles) in the region where the thermomechanical pumping was forced by the heater. The next step was to measure the ^3^$\hbox {He}$ concentrations simultaneously in two places: up and downstream of the filter. The obtained result is shown in Fig. 4b where the decrease of upstream concentration appeared simultaneously with a relatively strong increase of the downstream one. This behavior was opposite to previous observations of the operation of any entropy filter ever tested. The result in Fig. 4 suggests that standard quantum filtration/separation does not appear when the filter is made of a superconducting material. Same as before, the unexpected increase of the ^3^$\hbox {He}$ concentration occurring downstream of the filter, could be explained by the extreme difference in thermal conductivities of helium and YBCO-123 in the temperature region below $\lambda$-transition, which creates the rectification-like conditions. A deeper discussion is given below.

## Discussion: superconductivity versus superfluidity

The low thermal conductivity of YBCO-123 entropy filter is crucial for the explanation of the effects described above. Experimental results presented in papers^16,23,24^ show that thermal conductivity *k* of YBCO-123, in the vicinity of 2 K, is close to or even below $1 \hbox { Wm}^{-1}\hbox {K}^{-1}$. Especially important is the review paper by Uher^24^, which discusses different aspects of *k* of superconducting perovskites in general. From the data presented in this publication, we know that *k* values of these materials are in the range of $0.1 - 1 \hbox { Wm}^{-1}\hbox {K}^{-1}$ in this temperature region. We also attempted to estimate the *k* value of YBCO-123 entropy filter based on the data from our experiments.

**Fig. 5:**
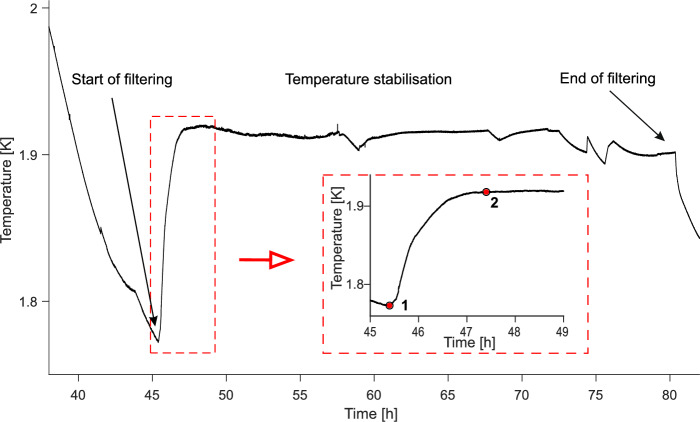
Temperature evolution below the $\lambda$-transition before and during the thermomechanical operation of the YBCO-123 entropy filter in superfluid helium. Insert shows the points 1 where the heater is on, and 2 where the system reaches equilibrium. These points were used to evaluate $\textit{k}_{x}$.

Figure 5 shows the $\sim 0.15$ K temperature “jump” (lasting about 2 h) which occurred in helium after switching the heater on. Knowing the heat supplied by the heater and calculating the enthalpy difference of the system at points 1 and 2 from the insert of Fig. 5, we could estimate the value of the thermal conductivity coefficient of the entropy filter. From the definition of thermal conductivity, we know that:

1 $$\begin{aligned} \frac{Q_{ef}}{t}=\dfrac{k_{x}S\Delta T}{\Delta l} \end{aligned}$$

where $\textit{k}_{x}$ is the desired thermal conductivity coefficient, *S* is the cross-section area of the filter, and $\Delta$*l* is its thickness. $\textit{Q}_{ef}$ is the heat absorbed by the entropy filter and in the time necessary to achieve equilibrium after switching the heater on, $\Delta$*T* is the temperature difference between points 1 and 2.

In general, heat absorbed by the entropy filter $\textit{Q}_{ef}$ is the difference between heat emitted by the heater ${Q}_{h}$ and that absorbed by liquid helium $Q_{LHe}$. Additional energies that need to be taken into account are: $\textit{Q}_{t}$ which is absorbed by the structural elements and $\textit{Q}_{f}$—the heat incoming from the outside. The whole process can be described by the Eq. (2):

2 $$\begin{aligned} Q_{ef}=Q_{h}-Q_{LHe}-(Q_{t}-Q_{f}) \end{aligned}$$

where the right side of Eq. (2) comprises: ${Q}_{h}$—the heat emitted by the heater (the driving force for thermomechanical pumping), $Q_{LHe}$—the change in internal energy of the liquid helium in the tank, $\textit{Q}_{t}$—the heat absorbed by structural elements of the tank (we assume that it can be neglected because it is transferred in the process of the lossless heat transport of superfluid helium^22^), and $\textit{Q}_{f}$—the heat potentially incoming from outside via the fountain—this can also be neglected because downstream helium is removed with the pumping system which rather leads to decreasing the temperature. Working above 1 K allows the Kapitza resistance to be ignored. Taking $\textit{Q}_{t}$ and $\textit{Q}_{f}$ as negligibly small makes it possible to estimate $\textit{k}_{x}$, since in this case $\textit{Q}_{ef}$ is equal only to the energy generated by the heater minus $Q_{LHe}$ calculated from the enthalpy difference of liquid helium at points 1 and 2 (see Fig. 5) using HEPAK v.3.4. The HEPAK code is based on a combination of two equations of state: one for superfluid and lambda lines^25^ and one for normal helium^26^, combined in a region of overlap at about 2.5 K in the liquid. From the equations of state, the code calculates the enthalpy, the accuracy of which was verified by CODATA^27^. After determining *Q*$_{ef} = 12.38(56)$ J from Eqs. (1) and (2) with ${Q}_{h} = 504$ J and *Q*$_{LHe} = 491.61(44)$ J, the thermal conductivity coefficient was estimated taking into account the following values: $\textit{S} = 1\times 10^{-4}$ $\hbox {m}^{2}$, $\Delta \textit{l} = 1\times 10^{-2}$ m, *t* = 7200 s, $T_{1}$ = 1.773 K, $T_{2}$ = 1.918 K, pressures 18.1 hPa and 28.53 hPa, volumes of liquid helium in the container 8.9(34) and 8.1(34) liters—taken from the weight measurements: 1.29(1) kg and 1.18(1) kg (points 1 and 2, insert of Fig. 5) with liquid helium density in vicinity of 2 K equal 0.145 kg/l^20^. Obtained result $\bf \bf \textit{k}_{x}$ $\bf \approx$** 1.18(64)** $\bf {\text{Wm}}^{-1}$**K**^−1^ is in good agreement with the data from literature^16,23,24^.

A special argument supporting the validity of our approach is the consistency with the results presented in Uher’s work^24^, which pointed out different aspects and similarities between various perovskites regarding their thermal conductivity below $\textit{T}_{c}$. Based on the data for ${\text{La}}_{2-x}$ ${\text{Sr}}_{x}$ ${\text{CuO}}_{4}$, Uher proposed a Debye model in which the mean free path for phonons, responsible for thermal transport in superconductors far below $\textit{T}_{c}$, is equal to the size of the grains. For unconnected superconducting grains, Uher obtained a grain size of about $10 \mu \hbox {m}$. This confirms our result given in Fig. 1, as well as the manufacturer data.

**Fig. 6:**
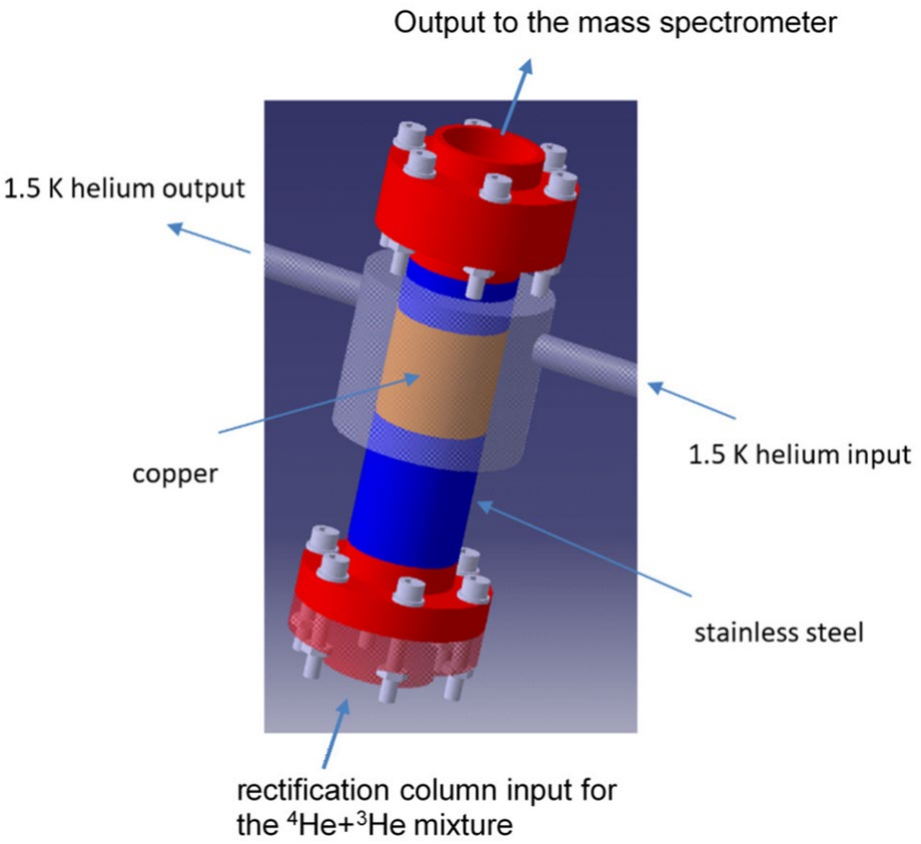
Schematic presentation of the rectification column. The temperature of the copper part is stabilized by the flowing liquid helium with the temperature at the level of 1.5 K. 3D model created in Autodesk Inventor Professional 2018 (https://www.autodesk.com/products/inventor/overview).

When helium temperature increases by 0.15 K, the entropy filter will still be at 1.7 K for some time because of its extremely low thermal conductivity (as well as thermal diffusivity, which also drops down below 20 K^28^). Thus, the temperature difference between the entropy filter (1.77 K) and superfluid helium (1.92 K) is maintained long enough to create conditions suitable for a rectification-like process, where the entropy filter would work similarly to a rectification column with YBCO-123 particles acting as rectification plates. The above explanation is evidenced by the change of ^3^$\hbox {He}$ concentration measured downstream, as shown in Fig. 4b. It starts to increase when the heater is on, and after some time it decreases despite the heater working. This behavior of ^3^$\hbox {He}$ concentration after about 50 h of the experiment indicates that temperature equalization in the system leads to the disappearance of the rectification-like effect when $\Delta$*T* reaches zero. It is also worth noting that the observed effect can be easily destroyed by overheating even at low power in the laminar flow regime. Moreover, the observed decrease of the ^3^$\hbox {He}$ concentration after reaching its maximum indicates the lack of Meissner effect (magnetic ”levitation”) of ^3^$\hbox {He}$ atoms (magnetic, due to 1/2 nuclear spin) between the superconducting grains. This space could form percolation paths for the free transfer of ^3^$\hbox {He}$ atoms through the filter, which was considered a possible mechanism for effective filtration. This couldn’t be confirmed at the current research stage. The above results motivated us to construct a small rectification column with YBCO-123 inside to test if it would work above the lambda point, for liquid helium in a normal state and the initial concentration of ^3^$\hbox {He}$ in the $^{4}\hbox {He}$+^3^$\hbox {He}$ mixture being as low as 0.2 ppm. The column mounted inside the prototype separator^29^ is shown in Fig. 6.

**Fig. 7:**
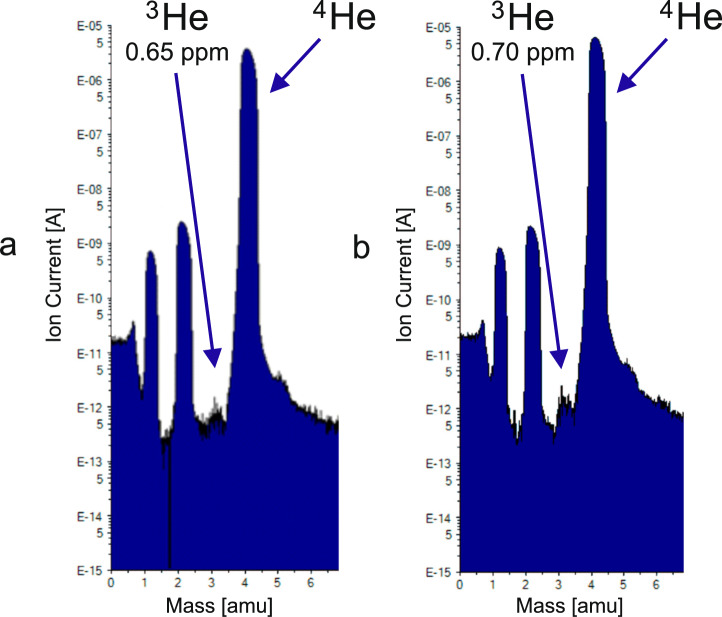
Mass spectra in two separated experiments, below (**a**) and above (**b**) $\lambda$-transition. Spectra were registered for the rectification column from Fig. 6: (**a**) mass spectrum for 1.93 K at the bottom of column and (**b**) mass spectrum for 3.3 K at the bottom of the column—the separation efficiency depends little on whether the rectification is below or above $\lambda$-transition. The presence of hydrogen peaks at masses 1 and 2 are explained in our previous paper^17^.

Rectification tests were performed both below and above the $\lambda$-transition. Results shown as mass spectrometer spectra are presented in Fig. 7.

## Conclusions

Operation of the *fountain effect* generated with the YBCO-123 entropy filter, unlike the other filters, allowed the observation of a strong increase of ^3^$\hbox {He}$ concentration downstream, where only pure $^{4}\hbox {He}$ was expected. This effect occurred due to the unique combination of two quantum phenomena—superfluidity and superconductivity, leading to the observation of a low-temperature rectification-like process. Initial results with the rectification column filled with YBCO-123 micro-powder confirm that using this process is a viable idea. Should it be confirmed in further experiments, it would give a unique opportunity to reduce the energy consumption, since in standard experiments with the entropy filter it is necessary to cool helium down below the $\lambda$-point, as well as to operate with this method outside the Earth—e.g. in space, where T = 2.7 K (temperature of the microwave relic radiation). It could make extraction of ^3^$\hbox {He}$ from the Moon’s regolith an economically viable method^3,4^. Low thermal conductivity materials are usually used as plates in rectification columns working at low temperatures. Hydrogen isotopes separation has to be performed below 20 K^30^, so our method could probably be used also to obtain deuterium, the next essential ingredient of the $\hbox {D}+^{3}\hbox {He}$ reaction.

## Supplementary Information

Supplementary Information 1.

Supplementary Information 2.

Supplementary Information 3.

## Data Availability

All data and their sources supporting the findings of this work will be available on request. Wojciech Kempiński (wojkem@ifmpan.poznan.pl) is the contact person.

## References

[1] Cho, A. Helium-3 shortage could put freeze on low-temperature research. *Science* **326**, 778–779. 10.1126/science.326_778 (2009). 19892947 10.1126/science.326_778

[2] Shea, D. & Morgan, D. The helium-3 shortage: Supply, demand, and options for congress. In *Congressional Research Service* R41419 (2011).

[3] Simko, T. & Gray, M. Lunar helium-3 fuel for nuclear fusion: Technology, economics, and resources. *World Future Rev.* **6**, 158–171. 10.1177/1946756714536142 (2014).

[4] Ding, C. et al. Moon-based ground penetrating radar derivation of the helium-3 reservoir in the regolith at the Chang’E-3 landing site. *IEEE J. Select. Top. Appl. Earth Observ. Remote Sens.* **16**, 2764–2776. 10.1109/JSTARS.2023.3253499 (2023).

[5] Kulcinski, G. L. & Santarius, J. F. Advanced fuels under debate. *Nature* **396**, 724–725. 10.1038/25456 (1998).

[6] Rostoker, N., Binderbauer, M. W. & Monkhorst, H. J. Colliding beam fusion reactor. *Science* **278**, 1419–1422. 10.1126/science.278.5342.1419 (1997). 9367946 10.1126/science.278.5342.1419

[7] Mazzucato, E. A first generation fusion reactor using the D-3He cycle. *Fusion Sci. Technol.* **77**, 1–7. 10.1080/15361055.2020.1858673 (2021).

[8] Stott, P. E. The feasibility of using D-3He and D-D fusion fuels. *Plasma Phys. Controlled Fusion* **47**, 1305. 10.1088/0741-3335/47/8/011 (2005).

[9] Tuszewski, M. Field reversed configurations. *Nucl. Fusion* **28**, 2033. 10.1088/0029-5515/28/11/008 (1988).

[10] Wittenberg, L. J. *Non-Lunar 3He Resources* (Second Wisconsin Symposium on Helium-3 and Fusion Power 19-21, Madison, WI, 1993). http://fti.neep.wisc.edu/pdf/fdm967.pdf.

[11] Uniper. Irkutsk oil and uniper have signed long-term helium sales and purchase agreement. Webpage (2020).

[12] Lang, M., Afanasyev, I., Slutskiy, B. & Schmid, F. Monetizing gas of a giant high helium and nitrogen gas reservoir—amur gas processing plant. *World Petroleum Congress*. https://onepetro.org/WPCONGRESS/proceedings-pdf/WPC22/3-WPC22/D033S013R004/1254864/wpc-22-1513.pdf (2017).

[13] Nakai, H., Kimura, N., Murakami, M., Haruyama, T. & Yamamotoa, A. Superfluid helium flow through porous media. *Cryogenics* **36**, 667–673. 10.1016/0011-2275(96)00030-6 (1996).

[14] Eselson, N. Raztwory kwantowych zidkostei He3–He4. *Nauka*10.1038/141913a0 (1973).

[15] Niechciał, J. et al. Separation of 3He isotope from liquid helium with the use of entropy filter composed of carbon nanotubes. *Energies*10.3390/en14206832 (2021).

[16] Morelli, D. T., Heremans, J. & Swets, D. E. Thermal conductivity of superconductive Y-Ba-Cu-O. *Phys. Rev. B* **36**, 3917–3919. 10.1103/PhysRevB.36.3917 (1987). 10.1103/physrevb.36.3917 9943337

[17] Kempiński, W. et al. Helium3 isotope separation and lambda front observation. *Sep. Purif. Technol.* **210**, 276–280. 10.1016/j.seppur.2018.08.003 (2019).

[18] Lane, C. T. *Superfluid Physics* (McGaw-Hill Book Company Inc., 1962).

[19] Enss, C. & Hunklinger, S. *Low-Temperature Physics* (Springer, 2005).

[20] Kempiński, W. *et al.* Bose-Einstein condensate—from superfluidity to superconductivity. In *Proceedings of the ICEC 23–ICMC 2010*, 35–40 (2011).

[21] Kuznetsov, V. M. Separation of helium isotopes by rectification and thermo-osmosis. *Soviet Phys. JETP* **5**, 819–827 (1957).

[22] Lantz, J. *Heat Transfer Correlations Between a Heated Surface and Liquid* & *Superfluid Helium: For Better Understanding of the Thermal Stability of the Superconducting Dipole Magnets in the LHC at CERN* (Linkopings Universitet, Thesis at Department of Management and Engineering, 2007).

[23] Bondarenko, A. V., Gavrenko, O. A., Merisov, B. A., Obolenskiǐ, M. A. & Sologubenko, A. V. Heat conductivity of YBa2Cu3O(7–x) single crystals in the range 2–300 K. *Fizika Nizkih Tiemperatur* **17**(3), 318–322 (1991).

[24] Uher, C. Thermal conductivity of high-Tc superconductors. *J. Supercond.* **3**, 337–389 (1990).

[25] Arp, V. D. State equation of liquid helium-4 from 0.8 to 2.5 K. *J. Low Temp. Phys.* **79**, 93–114. 10.1007/BF00683459 (1990).

[26] McCarty, R. D. & Arp, V. D. *A New Wide Range Equation of State for Helium, 1465–1475* (Springer, New York, 1990).

[27] Cox, J. D., Wagman, D. D. & Medvedev, V. CODATA key values for thermodynamics (1989).

[28] Fujishiro, H. et al. Anisotropic thermal diffusivity and conductivity of YBCO(123) and YBCO(211) mixed crystals. *I. Jpn. J. Appl. Phys.* **33**, 4965. 10.1143/JJAP.33.4965 (1994).

[29] Chorowski, M. et al. Continuous flow system for controlling phases separation near lambda transition. *AIP Conf. Proc.* **1573**, 276–284. 10.1063/1.4860712 (2014).

[30] Alekseev, I. et al. Cryogenic distillation facility for isotopic purification of protium and deuterium. *Rev. Sci. Instrum.* **86**, 125102. 10.1063/1.4936413 (2015). 26724068 10.1063/1.4936413

