# Supplementary material for: When superfluidity meets superconductivity in the extraction of 3He isotope from liquid helium
Source: Sci Rep. 2025 Jul 2;15:22822. doi: 10.1038/s41598-025-05542-8 (PMC12216688; doi:10.1038/s41598-025-05542-8)
Supplement: Supplementary file 3 — Supplementary Information 3. [file 41598_2025_5542_MOESM3_ESM.pdf]

Supplementary material

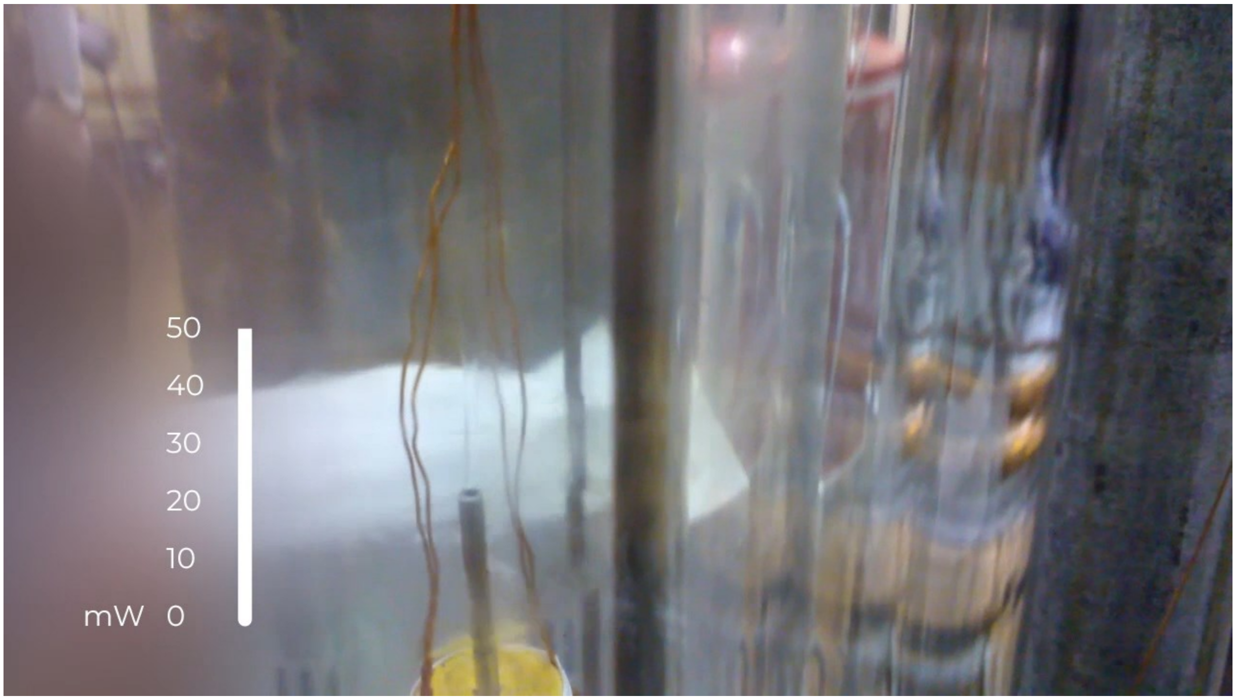

Movie 1. Thermomechanical effect (*fountain effect*). The experiment was performed in a laminar regime with the use of the insert shown in Fig. 2 without a pyrex part (marked "1" in Fig. 2b). Due to the low efficiency of the YBCO-123 entropy filter, the video shows the operation of an entropy filter made of MWCNTs.

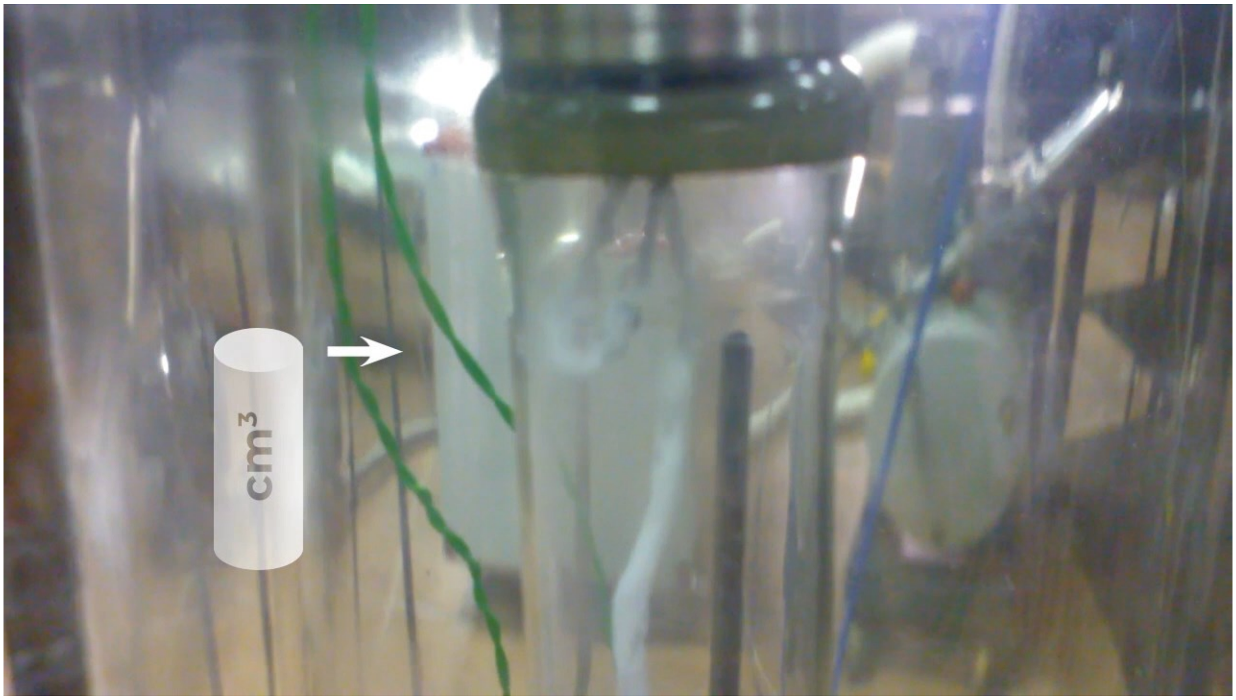

Movie 2. The efficiency of the filter as a volumetric flow vs time. The experiment was performed with the use of the insert shown in Fig. 2. Due to the low efficiency of the YBCO-123 entropy filter, the video shows the operation of an entropy filter made of MWCNTs.
